# 

**Published:** 1902-12

**Authors:** 


### MARRIAGE.

AT Kansas City, Mo., November 8, 1902, at the bedside of his dying father, Dr. Junius H. Wattles, Jr., was married to Miss Amy A. Bamber. The ministrations of the Rev. W. F. Richardson in binding together in matrimony the son and his chosen helpmate was concluded in a little while by the last ministrations to the father, whose last wish on earth was to witness the marriage of his son and to place upon them the parental blessing.

---

### NECROLOGY.

### PERSONALS.

Dr. Ira G. Coble, of Berwick, Pa., in connection with his practice conducts a large livery and boarding stable.

Dr. S. C. Babson, of Kennett Square, Pa., a graduate of the Veterinary Department, University of Pennsylvania, Class of 1902, is taking the Pasteur treatment for rabies incidental to an abrasion on the hand while holding an autopsy on a rabid dog.

The recognition of Dr. W. S. Kooker, of Philadelphia, and his election to the Presidency of the Keystone Veterinary Medical Association is a well-merited tribute to a faithful devotee of the profession.

Dr. J. C. Burneson, formerly of the Ohio Agricultural Experiment Station, Wooster, Ohio, is now a member of the Bureau of Animal Industry Inspection force at the G. H. Hammond Co., Hammond, Indiana.

Dr. H. A. Christman of the Bureau of Animal Industry, and located at Cincinnati, Ohio, was a visitor to Philadelphia early in October.

Dr. James Marshall, of Philadelphia, and W. L. Rhoads, of Lansdowne, Pa., were active in the Road Drivers' Parade in Philadelphia, in October.

Dr. I. W. Zellars, of Harrisburg, Pa., was a visitor to Philadelphia in October. Dr. Zellars is very much interested in the enforcement of the State laws regulating veterinary practice.

Dr. G. H. Welliver, of Bloomsburg, Pa., in connection with his practice conducts a large boarding and livery stable, and deals extensively in horses.

Dr. L. Van Es, of Mobile, Alabama, has accepted the chair of veterinary science at the North Dakota Agricultural College, and will enter upon his work there about January 1, 1903. Dr. Van Es is well equipped to fill the position to which he succeeds.

### INDEX.

ABNORMAL fœtus, an, 240
Abortion in cows, 280, 360
Abscess of the pleura, 110
of the spleen caused by swallowing a needle, 643
Acad. de Med., 367
de Sc. de Paris, 237, 296, 367, 429
Actinomycosis in a dog, 241
Action of water drunk during digestion, 370
Acute œdema of the tongue, 172
Adams, J. Dowling, 583
Address of the President, James Law, 606
of welcome by Hon. Mr. Young, 655
President's, Illinois State Veterinary Medical Association, 21
P. S. V. M. A., 224
Adenoma of the mammary glands, 164
A., E. B., 379
Afterbirth in cows, retained, 620
Agricultural Journal, 52, 580
Albuminuria and hæmaturia, renal colic with, 643
Alcohol upon the evolution of tuberculosis, influence of, 582
the gastric juice, action of, 238
Alimentary intoxication of the pig, 577
Allegheny County Veterinary Medical Association, 248, 263, 782
Alopecia in a cow, generalized, 720
Alsace, glanders in, 36
Alternating currents, 510
Alumni Society of the Veterinary Department of the University of Pennsylvania, 460
American Journal of the Medical Sciences, 582
Veterinary Medical Association, 130, 247, 314, 375, 442, 589, 592, 649, 652, 725, 730, 731, 778
appeal of Dr. J. G. Rutherford, 730
committees 1902–03, 649
resident State secretaries 1902–03, 731
what is the proper field of the, 120
Review, 45
Ames, Arthur V. S., necrology of, 178
Amnion, polyhydramnios, or dropsy of the, 40
Among the Board of Examiners, 724
the colleges, 54, 188, 446, 531, 650, 726
Amputation of a bull's penis, 302
Anæsthesia, local, 428
Analgesia, cocaine-morphine, 294
Anasarca, antistreptococcus serum in, 238
Anastomosis in the dog, a simple method of performing entero-enteral-, 160
Anatomo-pathological study of ringbone and spavin, 133
Anderson, Jno., marriage, 314
Anent "The Breeders' Gazette's" attitude toward the veterinary profession, 768
Animal, hermaphrodism in a bovine, 367
husbandry, report of the committee on, 387, 516
Animals, a plan for the disposal of tuberculous, 43
especially those of the bovine species, of the tubercle bacilli of bovine and human origin, comparative experiments on the pathogenic action for, 167
Annales de Medicine Veterinaire, 110, 167, 238, 239, 294, 368, 428, 506, 643, 718, 767
Anomaly of the kidneys, 643
Another danger signal out, 509
school in the field, 510
Annual meeting of the Iowa State Veterinary Medical Association, call for the fourteenth, 57
of the Veterinary Medical Association of New Jersey, 125
Another stone in the arch, 305
Ano-vulvitis, external ulcerative, 285, 546
Anthelmintic, benzine as an, 32
Anthelmintics, 583
Anthrax in a horse, 201
the value of co-operation in the sanitary control of our periodic epizootics of, 484
Antirabic treatment, 239
Antiseptic, formin as an intestinal, 641
Antistreptococcus serum in, 238
Antitoxin in distemper, 116
Antivenenes, snakes, snake venoms and, 343
Aphthous fever, chromic acid in, 35
Archiv Med. d'Hugers, 164
Arizona—New York, 646
results of strict sanitary regulations in, 613
Arthritic synovitis, creolin (Pearson) in, 116
Artiodactyla, syndactylism of the median digits in, 428
Arytenectomy, operative variation in, 297
two cases of, 165
successful cases of, 319
Asses' milk for infants, the use of, 35
Ass, properties and composition of the milk of the, 506
Association meetings, 376, 785
proceedings, 47
work, some, 175
Assurance society, an, 646
Atlas of clinical medicine, surgery and pathology—book review, 54
Atmospheric air for strained tendons, insufflation of, 296
Atrophic orchitis in a bull, chronic, 203
Atropine and morphine, strychnine-poisoning treated successfully with, 113
Attitude of the Dominion shorthorn breeders toward the tuberculin test, the, 118
of the farmer toward the tuberculin test, the, 90
Aull, Miss Clara F., marriage of, 724
Avery, S., 704
Avian diphtheria, 295, 369
A. V. M. A., Minneapolis, Minnesota, September, 1902, 375, 771, 778
the field of, 305

Azoturia, 111
rupture of the flexor tendons as a complication of, 303

BACILLUS from human and bovine sources, the comparative virulence of the tubercle, 65
Bacterial contamination of milk, 581
Baker, A. H., V.S , 562
Barley as a substitute for oats in the horse's rations, 298
Barron, Thos. F., V.S., necrology, 46
Baughman, D. E., M.D.C., 214
Beattie, S., secretary, 266, 663
Beatty, James, V.M.D., necrology, 47
Behring's recent announcement in regard to tuberculosis of cattle, 36
Bell, W. L., D.V.S., 170, 172
Benzine as an anthelmintic, 32
Berns, George H., D.V.S., 750
Berliner Thier Wochenschrift, 766
Berry, H. K., secretary, 729
Binding of the JOURNAL, 732
Bismuth salicylas, 583
Bitch, castration of the, 718
chronic galactophoritis in the, 575
Blastomyces (Torula), a tumor-like lesion in lung of a horse caused by a, 740
Blister, a new, 238
Blood of epileptic persons, parasites in the, 423
serum of the horse in laparotomies, 505
Board of Examiners, among the, 724
of Health of the City of Nashville, municipal meat-inspection legislation, with special reference to the law and the rules and regulations of the, 6
trials of the veterinary examining, 287
Boastfulness as an evidence of inner weakness, 723
Boil, etiology of shoe, 688
Book news, 531
review, 54
Boon, George M., V.S., 433, 584
Boston horseshow, veterinary inspection at the, 189
Bovine animal, hermaphrodism in a, 367
sources, the comparative virulence of the tubercle bacillus from human and, 138
tuberculosis to human beings, the final overthrow of Koch's theory respecting the non-communicability of, 586
Bovines, puerperal mania in, 571
Boyd, Chas W., V.M.D.. 303
Boyer, Miss Myrtle, marriage, 314
Breeders toward tuberculin test, the attitude of the Dominion shorthorn, 118
Brimhall, S. D., 261
British Medical Journal, 581
Bull, chronic atrophic orchitis in a, 203
Bull. de la Soc. Cent. de Méd. Vet., 238
Bull. Gen. de Therapeut., 641
Bulletin Veterinaire, 32, 35
Bull, situs transversus in a, 298
Bull's penis, amputation of a, 302
Bureau, Massachusetts new, 176
Bursattee, treatment of malignant œdema with carbolic acid, with remarks upon, "leeches" or, 301
Butler, Tait, V.S., 498, 601
Butterfield, J. F., V.S., 275

CÆSAREAN section, 283
Calculi, 275
urethral, 115
Calculus, urethral, 205
Calorimeter for studying the nutrition of man, the improved respiration, 186
Calves and Pasteurized milk, 51
Cancer from man to the white rat, inoculation of, 504
new microbe of, 504
Cancers, parasitic theories of, 505
Canina, piroplasma, 33
Canine distemper, vaccination against, 506
instrument, a new, 293
and feline instrument, a new, 293
medicines, new, 583
Carcinoma, the pathology of, 580
Carbolic acid, treatment of malignant œdema with, 301
Carl, L. W., 246
Carnegie's munificence, 61
Carrick, C. J., necrology, 248
Carter, J. M., V.M.D., 337
Cases, a report of three interesting, 433
parturition, 211
Castration of the bitch, 718
Catarrh of the newborn, raw milk in marasmus and intestinal, 32
Cattle against tuberculosis, some experiments upon the immunization of, 671
an enzootic attack of chorea among, 37
a new plan for controlling tuberculosis of, 16
a new trypanozoma in, 423
Behring's recent announcement in regard to tuberculosis of, 36
external ulcerative anovulvitis of, 546
phytolacca poison in, 439
Texas fever in native South Carolina, 81
the X-ray as an aid in the diagnosis of tuberculosis in, 97
transmissibility of human tuberculosis to, 367
Cerebral hemorrhages in the horse, multiple, 719
Chicago Veterinary College, commencement exercises, 383
Chief of the Bureau of Animal Industry, wishes for, 647
China, German meat-inspection in, 36
Chorea among cattle, an enzootic attack of, 37
Chromic acid in aphthous fever, 35
Chronic atrophic orchitis in a bull, 203
galactophoritis in the bitch, 575
Chrysoform, 368
City's milk supply, some thoughts on a, 352
Civil-service examinations, 123
Clark, W. G., secretary, 265
Clement, A. W., Ohio's tribute to the late Dr., 247
Clinical Medicine, Surgery and Pathology, an Atlas of, book review, 54
notes on the treatment of various diseases in dogs, 429
Clitoridectomy for incontinence of urine, 162
Clysters of hydrogen peroxide, 294
Cocaine-morphine analgesia, 294
Cold in the etiogenesis of pneumonia, 234
"Cold" shoulder lameness, treatment of, 165
Colic with albuminuria and hæmaturia, renal, 643
Colleges, among the, 54, 188, 446, 531
Colon of a horse, impaction of the fourth fold of the large, 436
Columella, 16
Columella's plan for controlling tuberculosis, regarding, 312
proposition, 175, 245
for the control of tuberculosis, discussion of 179, 249
Commencement exercises:
Chicago Veterinary College, 383
Grand Rapids Veterinary College, 318
Kansas City Veterinary College, 315
McGill University, veterinary department, 316
McKillip Veterinary College, 317
New York-American Veterinary College, 530
Ontario Veterinary College, 380
Veterinary Department, University of Pennsylvania, 445
Western Veterinary College, 315

Comments on the death of Dr. Huidekoper, 44, 178
Committee on animal husbandry, report of the, 387, 515
on diseases and treatment, report of the, 330
on intelligence and education, report of the, 387, 447
on legislation, report of the, 457
on sanitary science and police, report of the, 513
on sanitation, report of the, 321
reports, Pennsylvania State Veterinary Medical Association, 386
Committees, Pennsylvania State Veterinary Medical Association 1902–03, 458
Communications, 48, 124, 179, 377, 778
Company, a Japanese dairy, 53
Comparative experiments on the pathogenic action for animals, especially those of the bovine species, of the tubercle bacilli of bovine and human origin, 167
virulence of the tubercle bacillus from human and bovine sources, 65, 138
Composition of milk of the ass, properties and, 506
Complication of azoturia, rupture of the flexor tendons as a, 303
Comp.-rend. Acad. des Sci., Paris, 237, 296, 423
Comstock, John R., V.S., necrology, 314
Condition of neoplasia exhibiting progressive malignancy, 291
Connecticut criticises "Columella," 181
Veterinary Medical Association, 194. 729
Contagious diseases, State control of, 324
Contamination of milk, bacterial, 581
Contribution to the study of immunity to tuberculosis, Pennsylvania's, 722
Contrivance for the ready handling of disabled horses, a, 25
Controlling tuberculosis of cattle conservatively, a proposal of a new plan, 16
Conveyer of diseases, milk as, 762
Cooper, T. J., 762
Co-operation in the sanitary control of our periodic epizootics of anthrax, the value of, 484
Corcoran, R. B., veterinarian, 45
Cord, experiments upon the excitability of the spinal, 429
Corneal ulcers, treatment of, 718
Correction, 122
Correspondence, 49, 183, 249, 312
Corrosive sublimate in foot-and-mouth disease, 371, 642
intravenous injections of, 166
Cow case, a, 207
cutaneous lymphadenoma in a, 295, 427
on stenosis of the duct in the teat of the, 574
retained afterbirth in, 620
generalized alopecia in a, 720
some diseases incidental to pregnancy and parturition in the, 630
Cows, abortion in, 280, 360
Creolin (Pearson) in arthritic synovitis, 116
Cressy, Noah, M.D., Ph.D., D.V.S., necrology, 588
Crichton, A. M., M.R.C.V.S., 630
Cricotomy for roaring, 239
Crossman, George S., V.S., necrology, 123
Crowforth, Anderson, secretary, 264, 530
Crystalline lens, luxation of the, 368
Cutaneous lymphadenoma in a, 427
Cyst of the great omentum, dermoid, 32
of the knee in the horse, 111

Dairy and milk-inspection, 323
company, a Japanese, 53
Dalrymple, W.H., M.R.C.V.S., 484
Dawson, Charles F., M.D., D.V.S., 301, 507
Day, Holman F., 26
Death of a hyena at the National Zoological Park, report on the, 424
of Dr. Huidekoper, comments on the, 44, 178
Deaths of gastric origin, sudden, 367
"December's garnering," 43
Dentistry, veterinary, 757
Department of canine, feline, and avian surgery:
Canine instrument, a new, 235
Chronic galactophoritis in the bitch, 575
Condition of neoplasia exhibiting progressive malignancy, a, 291
Entero-enteral anastomosis in the dog, a simple method of performing, 160
Goitre cured by iodine medication, 366
Hyena at the National Zoological Park, report on the death of, 424
Mange, relative susceptibility to follicular, 365
remedy for circumscribed follicular, 366
Maternal instinct, unusual exhibition of the, 366
Neoplasia exhibiting progressive malignancy, a condition of, 291
Relative susceptibility to follicular mange, 365
Report on the death of a hyena at the National Zoological Park, 424
Department of surgery:
Hoof diseases, 28
Dermoid cyst of the great omentum, 32
Dermosapol, 428
Deutsche Thierarztliche Wochenschrift, 36, 113
Deviation from the orthodox method of treating parturient paresis, and its etiology, a, 405
Diagnosis of foreign bodies in the œsophagus, 371
of tuberculosis in cattle, the X-ray as an aid in the, 97
Digestion, action of water drunk during, 370
Digital artery in founder, ligaturing of the, 369
Dilatation, iodine treatment, tendinous synovial, 373
Diphtheria, avian, 295, 369
Disabled horses, a contrivance for the ready handling of, 25
Discussion of Columella's proposition, 179, 249
Disease, corrosive sublimate in foot-and-mouth, 371
intravenous injections of corrosive sublimate in foot-and-mouth, 166
milk as a conveyer of, 762
treatment of foot-and-mouth, 297
Diseases, hoof, 28
incidental to pregnancy and parturition, some, 630
in dogs, clinical notes on the treatment of various, 429
Disposal of tuberculous animals, a plan for the, 43
Distemper, antitoxin in, 116
trichloride of iodine in, 319
Dog, actinomycosis in a, 241
a case of hæmatic filariasis in a, 507
a simple method of performing entero-enteral anastomosis in the, 160
meningitis in the, 720
mumps in the, 165
the, 446
tick disease in the, 33
Dogs, clinical notes on the treatment of various diseases in, 429
the failure of thymol to expel whipworms in, 733
Dominion shorthorn breeders toward the tuberculin test, the attitude of the, 118
Dow, B. K., secretary, 195, 730
Draught-horse, the points of a, 31
Drinkwater, Wm., V.S., 211
Dropsy of the amnion, polyhydramnios or, 40

Duct in the teat of the cow, on stenosis of the, 574
Duncan, J. T., M.D., V.S., 274

EDITORIAL ethics, 374
Editorials:
A. V. M. A., 771
Alternating currents, 510
American Veterinary Medical Association at Ottawa, 772
American Veterinary Medical Association, the field of the, 305
American Veterinary Medical Association, what is the proper field of the, 120
Anent "The Breeders' Gazette's" attitude toward the veterinary profession, 768
Animals, a plan for the disposal of tuberculous, 43
Another danger signal out, 509
Another school in the field, 510
Another stone in the arch, 305
Arizona, New York, 646
Association work, some, 175
Assurance society, an, 646
At it again, 122
Attitude of the Dominion shorthorn breeders toward the tuberculin test, 118
Boastfulness as an evidence of inner weakness, 723
Chief of the Bureau of Animal Industry, wishes for, 647
Columella's proposition, 175, 245
Death of Dr. Junius Wattles, Sr., 772
"December garnerings," 43
Dominion shorthorn breeders toward the tuberculin test, the attitude of the, 118
Disposal of tuberculous animals, a plan for the, 43
Editorial ethics, 374
Editorial strabismus, 43
Field of the A. V. M. A., the, 305
Frontispiece, our, 442
Journal, 1902, 773
Koch's theory respecting the non-communicability of bovine tuberculosis to human beings, the final overthrow of, 586
Magnanimous gift, 375
Massachusetts' new bureau, 176
Minneapolis in September, 1902, 374
Minneapolis wins, 121
Penalty of neglecting veterinary medicine, the, 508
Pennsylvania's contribution to the study of immunity to tuberculosis, 722
President's address, the, 586
Quarter of a century's usefulness, a, 245
Secretaryships, 176
State greetings, 647
The Veterinarian and the Journal of Comparative Pathology and Therapeutics, 773
Tuberculin test, the attitude of the Dominion shorthorn breeders toward the, 118
What is the proper field for the American Veterinary Medical Association? 120
Where in 1902? 42
Will Ottawa Aid the Movement? 772
Editorial strabismus, 42
Ectropion and entropion, 501
Edwards, Loxla, D.V.S.—necrology, 177
Eger, Alex., 46
Ellis, R. W., 757
Entero-enteral anastomosis in the dog, a simple way of performing, 160
Enzootic attack of chorea among cattle, an, 37
Epileptic persons, parasites in the blood of, 423
Epizootics of anthrax, the value of co-operation in the sanitary control of our periodic, 484
Equine pneumonic emphysema, the pathogenesis of, 562
Etiogenesis of pneumonia, cold in the, 234
Etiology of laminitis, 290
of rinderpest, 364
of shoe boil, 688
Etiquette, professional, 694
Evidence of inner weakness, boastfulness as an, 728
Examiners, New State Board of, 312
Examination of pathological specimens, anatomo-pathological study of ringbone and spavin as indicated by, 133
Exchange, 53
Exhibitions of the maternal instinct, unusual, 366
Exocardia, 644
Experiments upon the excitability of the spinal cord, 429
upon the immunization of cattle against tuberculosis, 671
External ulcerative ano-vulvitis, 285, 546
Exposition of hygienic milk supply, 784
Extracts from foreign journals (French and German):
Abscess of the pleura, 110
of the spleen caused by swallowing a needle, 643
Action of water drunk during digestion, 370
Adenoma of the mammary glands, 164
Alcohol upon the gastric juice, action of, 238
Alsace, glanders in, 36
Anæsthesia, local, 428
Antirabic treatment, 239
Anomaly of the kidneys, 643
Antistreptococcus serum in anasarca, 238
Artery in founder, ligaturing of the digital, 369
Artiodactyla, syndactylism of the median digits in, 428
Arytenectomy, operative variation in, 297
two cases of, 165
Ass, properties and composition of the milk of the, 506
Avian diphtheria, 295, 369
Azoturia, 111
Barley as a substitute for oats in the horse's ration, 298
Behring's recent announcement in regard to tuberculosis of cattle, 36
Benzine as an anthelmintic, 32
Bitch, castration of the, 718
Blister, a new, 238
Blood serum of the horse in laparotomies, 505
Botromycosis in the mammary glands of a mare, 766
Bovine and human tuberculosis identical, is, 113
Bull, situs transversus in a, 298
Cacodylates in malarial fever, the, 296
Cancer, new microbe of, 504
Cancers, parasitic theories of, 505
Canina, piroplasma, 33
Catarrh of the newborn, raw milk in marasmus and intestinal, 32
Cattle, transmissibility of human tuberculosis to, 367
China, German meat-inspection in, 36
Chromic acid in aphthous fever, 35
Chrysoform, 368
Clitoridectomy for incontinence of urine, 164
Cocaine-morphine analgesia, 294
Comparative experiments on the pathogenic action for animals, especially those of the bovine species, of the tubercle bacilli for bovine and human origin, 167
Consolidated fracture of the second phalanx following neurectomy, 764

Extracts from foreign journals (French and German):
Cow, generalized alopecia in a, 720
Corrosive sublimate in foot-and-mouth disease, 642
intravenous injections of, 166, 371
Cricotomy for roaring, 239
Cryptorchidism, observations upon, 111
Crystalline lens, luxation of the, 368
Cutaneous lymphadenoma in a cow, 295
Cyst of the great omentum, dermoid, 32
of the knee in the horse, synovial, 111
Dermoid cyst of the great omentum, 32
Dermosapol, 428
Diagnosis of foreign bodies in the œsophagus, 371
Diphtheria, avian, 295
Dog, meningitis in the, 720
mumps in the, 165
Dogs, clinical notes on the treatment of various diseases in, 429
Exocardia, 644
Fever, chromic acid in aphthous, 35
Fistula of the poll from foreign body, 719
Flexor tenotomy for knuckling, median neurectomy and, 109
Foal, pyæmia in the, 237
Foot-and-mouth disease, corrosive sublimate in, 642
Founder, osteitis of, 110
Fracture of the glenoid fibrocartilage of the second phalanx, 296
Gastric juice, action of alcohol upon the, 238
origin, sudden deaths of, 367
Gastro-intestinal strongylosis in ruminants, 504
German meat-inspection in China, 36
Gestation, vaginal, 238
Glanders in Alsace, 36
remarks on the sero-diagnosis of, 644
Glands, adenoma of the mammary, 164
Hæmoglobinuria of muscular origin, 720
treatment of, 111
Heifer to man, transmission of warts from the, 33
Hermaphrodism in a bovine animal, 367
Honthin, 429
Horse, frequency of tumors in the, 111
jabot of the jugular vein in the, 35
multiple cerebral hemorrhages in the, 719
synovial cyst of the knee of the, 111
tuberculosis in the, 32
Human tuberculosis to cattle, transmissibility of, 367
Humerus, tuberculosis of the, 32
Hydrogen peroxide, clysters of, 294
Iodide of potash in the treatment of lameness, 718
Incontinence of urine, clitoridectomy for, 164
Infants, the use of asses' milk for, 35
Injections of corrosive sublimate in foot-and-mouth disease, intravenous, 166
Inoculation of cancer from man to the white rat, 504
Insufflation of atmospheric air for strained tendons, 296
Iodine in the leucocytes, 237
Jabot of the jugular vein in the horse, 35
Juice of the sympathetic system, opotherapy with the, 113
Knee in the horse, synovial cyst of the, 111
Knuckling, median neurectomy and flexor tenotomy for, 109
Leucocytes, iodine in the, 237
Ligaturing of the digital artery in founder, 369
Luxation of the crystalline lens, 368
Malarial fever, the cacodylates in, 296
Mange, 34

Extracts from foreign journals (French and German):
Marasmus): and intestinal catarrh of the newborn, raw milk in, 32
Meat-inspection in China, German, 36
Mechanism of the pathogenic action of some helminths, 642
Median neurectomy and flexor tenotomy for knuckling, 109
Meningitis in the dog, 720
Morphine, strychnine poisoning treated successfully with atropine and, 113
Mumps in the dog, 165
Neurectomy and flexor tenotomy for knuckling, median, 109
operative modification in, 109
Observations upon cryptorchidism, 111
Œdema, 34
Œsophagus, diagnosis of foreign bodies in the, 371
Omentum, dermoid cyst of the great, 32
Operative modification in neurectomy, 109
variation in arytenectomy, 297
Opotherapy with the juice of the sympathetic system, 113
Osteitis of founder, 110
Pathological importance of the larvæ of the gastrophilus in the stomach of the horse, 766
Peroneal and tibial neurectomy in spavin, 297
Piroplasma canina, 33
Pleura, abscess of the, 110
Poisoning treated successfully with atropine and morphine, strychnine, 113
Pulmonary nodes in the lung simulating glanders in a horse affected with summer wounds, 294
Pyæmia in the foal, 237
Pyramidon, 164
Raw milk in marasmus and intestinal catarrh of the newborn, 32
Renal colic with albuminuria and hæmaturia, 643
Remarks on the sero-diagnosis of glanders, 644
Resection of the trachea, suturing and, 33
Roaring, cricotomy for, 239
Simple treatment for parturient fever, 774
Situs transversus in a bull, 298
Spavin, peroneal and tibial neurectomy in, 297
Spinal cord, experiments upon the excitability of the, 429
Strychnine poisoning treated successfully with atropine and morphine, 113
Suturing and resection of the trachea, 33
Sympathetic system, opotherapy with the juice of the, 113
Synovial cyst of the knee in the horse, 111
Tapeworm, toxin of the, 644
Tick disease in the dog, 33
Toxin of the tapeworm, 644
Trachea, suturing and resection of the, 33
Transmission of tuberculosis to man by accidental inoculation and experimental reinoculation of a calf, 765
of warts from the heifer to man, 33
Treatment of "cold" shoulder lameness, 165
of corneal ulcers, 718
of foot-and-mouth disease, 297
of hæmoglobinuria, 111
Trichorrhexis, 35
Tuberculosis in the horse, 32
of cattle, Behring's recent announcement in regard to, 36
of the humerus, 32
Tumors in the horse, frequency of, 111
Vaccination against canine distemper, 506
Warts from the heifer to man, transmission of, 33

FAILURE of thymol to expel whipworms from dogs, the, 733
Faivre, Dr. Charles—necrology, 444
Farm animals, the value of, 190
Farmer toward the tuberculin test, the attitude of the, 90
Farm, trouble on the, 26
Faust, John V. S., 587
Feeding on sinapis nigra, poisoning by, 242
Feline instrument, a new canine and, 293
Fever and its relation to the livestock interests of Tennessee, 625
chromic acid in aphthous, 35
in a horse, typhoid, 210
in native South Carolina, 81
the cacodylates in malarial, 296
the unsolved problems of milk, 230
Field of the A. V. M. A., 305
what is the proper? 120
Filariasis in a dog, a case of hæmatic, 507
Final overthrow of Koch's theory respecting the non-communicability of bovine tuberculosis to human beings, 586
Fisher, Adam, 178
Fistula of the poll from foreign body, 719
Flexor tendons as a complication of azoturia, rupture of the, 303
tenotomy for knuckling, median neurectomy and, 109
Fl. W. Milchhyg, 298
Foal, pyæmia in the, 237
Fœtus, an abnormal, 240
Follicular mange, ready for circumscribed, 366
Foot-and-mouth disease, corrosive sublimate in, 371
intravenous injections of corrosive sublimate in, 166, 642
treatment of, 297
warning to all owners of cattle, sheep, and swine, 775
Foot, the horse's, 156
Foreign bodies in the œsophagus, diagnosis of, 371
Formin as an intestinal antiseptic, 641
Foster, J. P , V.S., 50
Founder, ligaturing of the digital artery in, 369
osteitis of, 110
Fourteenth annual meeting of the Iowa State Veterinary Medical Association, call for the, 57
official report of, 251
Fracture of the glenoid fibrocartilage of the second phalanx, 296
Frank, E. T., M.D.C., 261
Fredericks, Wm. J., V.S., 729
French, Cecil, D.V.S., 160, 235, 291, 365, 424, 575
Frinck, J. H., V.S., 372
Frontispiece, our, 442
Frothingham, Langdon, M.D.V., 740

GALACTOPHORITIS in the bitch, chronic, 575
"Garnering, December's," 43
Gastric juice, action of alcohol upon the, 238
origin, sudden deaths of, 367
Gastro-intestinal strongylosis in ruminants, 504
Gastrophilus in the stomach of the horse, pathological importance of the larvæ of the, 766
Gay, Carl W., D.V.S., 90
Genesee Valley Veterinary Medical Society, 193, 529
German meat-inspection in China, 36
Gestation, vaginal, 238
Gibson, Geo. A., 329
Giffen, M. A., secretary, 398
Gift, a magnanimous, 375
Gillian, H. M., 326
Gilliland, S. H., V.M.D., 671
Glanders in Alsace, 36
in a horse affected with summer wounds, pulmonary nodes in the lung simulating, 294
remarks on the sero-diagnosis of, 644
Glands, adenoma of the mammary, 164
Glenoid fibro-cartilage of the second phalanx, fracture of the, 296
Glycosuria of muscular origin, 503
Goat's milk, 577
Gobeaud, G. J., D.V.S., 688
Gohn, H. W., V.S., 241
Goitre, cured by iodine medication, 366
Graham, Orr, V.S., 172
Grand Rapids Veterinary College, 318, 446
Grant, J. S., V.S., 178
Gray, Whitefield, secretary, 399
Greetings, State, 647
Gribble, Wm. H., secretary, 246
Griffith, Frederick, 25
Gunning, I. J., V.S., 21

HÆMATIC filariasis in a dog, a case of, 507
Hæmaturia, renal colic with albuminuria and, 643
Hæmoglobinuria of muscular origin, 720
treatment of, 111
Hagyard, John R., V.S., 50
Hail the microbe, all, 464
Hamilton, W., V.S., 201
Harger, S. J. J., V.M.D., 32, 109, 133, 164, 224, 294, 428, 504, 642, 718, 765
Harker, G. F., 126
Harness, injuries caused by ill-fitting saddlery and, 697
Harrington, Edw. T., V.S., secretary, 399
Harrison, R. H., D.V.S., 178
Hawkins, M. M., 648
Health, progress in veterinary medicine in its relation to public, 104
Heide, Dr. J. C., 178
Heifer to man, transmission of warts from the, 33
Helmer, Jacob, D.V.S., 447
Helminths, mechanism of the pathogenic action of, 642
Hemiplegia laryngis, investigation of the pathogenesis of roaring, 469
Hemorrhagica, purpura, 441
Hermaphrodism in a bovine animal, 367
Hints in pathology, practical, 228
Hoard's dairyman, 53
Honthin, 429
Hoof disease, 28
Horse, anthrax in a, 201
frequency of tumors in the, 111
impaction of the fourth fold of the large colon of a, 436
jabot of the jugular vein in the, 35
in laparotomies, blood serum of the, 505
multiple cerebral hemorrhages in the, 719
sickness, 356
synovial cyst of the knee in the, 111
the, 174
the points of a draught, 31
the weight of the kidneys of the, 274
tuberculosis in the, 32
typhoid fever in the, 210
Uncle Henry on the passing of the, 510
Horses, a contrivance for the ready handling of disabled, 25
molasses as a food for, 750
Horse's foot, the, 156
ration, barley as a substitute for oats in the, 298
Horseshow, veterinary inspection at the, 189
Hoskins, W. Horace, secretary, 192, 264, 311, 458, 651, 781
Howe, Wm. R., 247
Huidekoper, R. S., comments on the death of, 44, 178

Human and bovine sources, the comparative virulence of the tubercle bacilli from, 65, 138
tuberculosis to cattle, transmissibility of, 367
Humerus, tuberculosis of the, 32
Hutcheon, D., Col. Veterinary Surgeon, 580
Hyde, Andrew, President, 195
Hydrogen peroxide, clysters of, 294
Hydrophobia scares, 465
Hygienic milk supply, exposition of, 784
Hyena at the national zoological gardens, a report on the death of, 424
Hypertrophy of the heart, 641

ILLINOIS State Veterinary Medical Association, 321, 783
President's address, 21
Veterinary Medical and Surgical Association, 192, 527, 785
Immunization of cattle against tuberculosis, some experiments upon the, 671
Immunity to tuberculosis, Pennsylvania's contribution to the study of, 722
Impaction of the fourth fold of the large colon of a horse complicated by strangulation of the ileum in the foramen of Winslow, fatal, 436
Improved respiration calorimeter for studying the nutrition of man, the, 166
Incontinence of urine, clitoridectomy for, 164
Indiana State Veterinary Association, 258
Veterinary College, 446
Infants, the use of asses' milk for, 35
Influence of alcohol upon the evolution of tuberculosis, 582
Injections of corrosive sublimate in foot-and-mouth disease, intravenous, 166
Injuries caused by ill-fitting saddlery and harness, 697
Inoculation of cancer from man to the white rat, 504
Inspection at the Boston horseshow, veterinary, 189
Instrument, a new canine, 235
Insufflation of atmospheric air for strained tendons, 296
Intelligence and education, report of committee on, 387, 447
Interesting peculiarity, an, 377
Interest to you, matters of, 189
Interstate association of sanitary boards, 648
Intestinal catarrh of the newborn, raw milk in marasmus and, 32
Intoxication of the pig, alimentary, 577
Intravenous injections of corrosive sublimate in foot-and-mouth disease, 166
Investigation of the pathogenesis of roaring (hemiplegia laryngis), 469
Iodide of potash in the treatment of lameness, 718
Iodine in distemper, trichloride of, 319
in the leucocytes, 237
medication, goitre cured by, 366
treatment, tendinous synovial dilatations, 373
Iowa State Veterinary Medical Association, call for the fourteenth annual meeting of, 57
official report of the proceedings of the fourteenth annual meeting, 251, 321
Association, President's address, 407

JABOT of the jugular vein in the horse, 35
Jacob, M., V.M.D., 46
Japanese dairy company, a, 53
Jersey bulletin, 671
Jobson, Geo. B., V.S., 515
Joint, open, 208
Joke in the war office, a, 52
Jones, Lindsay, V.S., 178
JOURNAL, binding of the, 732
de Medecine Veterinaire de Lyon, 113, 577
et de Zootschnie, 111
Jugular vein in the horse, jabot of the, 35
Juice action of alcohol upon the gastric, 238
of the sympathetic system, opotherapy with the, 113

KANSAS CITY Veterinary College, 315, 463, 726
Kashiyyama Milk & Co., 53
Kaupp, B. F., secretary, 592
Keystone Veterinary Medical Association, 55, 129, 263
Kidneys, anomaly of the, 643
of the horse, the weight of the, 274
King, A. H., V.S., necrology, 123
Kinnel, G. W., 378
Kitchen, J. M. W., M.D., 230
Knee in the horse, synovial cyst of the, 111
Knowles, A.D., V.S., 37
Knuckling, median neurectomy and flexor tenotomy for, 109
Koch, reply of Professor E. Nocard to Professor, 1
Koch's theory respecting the non-communicability of bovine tuberculosis to human beings, the final overthrow of, 586
Koto, P. O., M.D.C., 407

LABORATORY notes on poison in sorghum, 704
La Clinica Veterinaria, 113, 166
Laddey, J. V., D.V.S., 97, 127
Lameness, iodide of potash in the treatment of, 718
treatment of "cold" shoulder, 165
Laminitis, etiology of, 290
Laparotomies, blood serum of the horse in, 505
La Presse Méd., 165, 295
La Semaine Médicale, 168
Law, Prof. Jas., V.S., 180, 606, 658, 711
L'Echo Vet., 774
Lecithin, therapeutic use of, 236
Legislation, 244, 307, 457
municipal meat-inspection, 6
report of the committee on, 457
Lennon, Joseph F., M.D.C., necrology, 588
Leucocytes, iodine in the, 237
Leuco-encephalitis in a horse, with positive results, notes on a feeding experiment to produce, 498
Ligaturing of the digital artery in founder, 369
Local anæsthesia, 428
Lowe, J. Payne, D.V.S., 127, 694
Lowe, Wm. Herbert, 104, 527, 729
Luff, J. H., Ph.G., V.S , 240
Luxation of the crystalline lens, 368
Lyman, Richard P., M.D.V., 183
Lymphadenoma in a cow, cutaneous, 295, 427

MACHAN, A., 664, 782
Machan, David, secretary, 527
Malarial fever, the cacodylates in, 296
Malcolm, Peter, V.S., 280
Mal de caderas, Voge's description of, 565
Mallein, 414
Magnanimous gift, a, 375
Mammary glands, adenoma of the, 164
Mange, 34
relative susceptibility to follicular, 365
remedy for circumscribed follicular, 366
observations on the treatment of scratches and, 584
Mania in bovines, puerperal, 571
Man, the improved respiration calorimeter for studying the nutrition, 186
Manitoba, the Veterinary Association of, 195

Man to the pig, transmission of tuberculosis from, 578
to the white rat, inoculation of cancer from, 504
Marasmus and intestinal catarrh of the newborn, raw milk in, 32
Mare, botryomycosis in the mammary gland of a, 766
Marriages, 50, 314, 724, 773
Marshall, C. J., V.M.D., 393, 513, 599, 670
Marten, E. G., M D.C., 205
Maryland State Veterinary Medical Society, resolutions of, 47
Masgana, George, V.S., 116
Massachusetts' new bureau, 176
Veterinary Association, 398
Massie, J., Veterinary Major, 221
Maternal instinct, unusual exhibition of the, 366
Matters of interest to you, 189
McDonough, James T., V.S., 156
McGill University, 316, 726
McKenzie, K. J., Secretary, 263
McKillip Veterinary College, 317
McKim, C., M.D.C., 419
McLeod, J. H., 331
Meat-inspection in China, German, 36
Median digits in artiodactyla, syndactylism of the, 428
neurectomy and flexor tenotomy for knuckling, 109
Medical Times, 577
Medicine in its relation to public health, progress in veterinary, 104
the new era of veterinary, 711
Meningitis in the dog, 720
Michigan State Veterinary Medical Association, 396
Microbe, all hail the, 464
of cancer, new, 504
Miles, Tarlton C., V.S., 248
Milk, bacterial contamination of, 581
as a conveyer of disease, 762
calves and Pasteurized, 51
fever cases, physicking in, 399
the unsolved problems of, 230
for infants, the use of asses', 35
goats', 577
-inspection, dairy and, 323
of the ass, properties and composition of the, 506
supply, some thoughts on a city's, 352
Miller, J., 326
Miller, S. T., D.V.S., 285
Miller, W. T., V.S., necrology, 510
Minneapolis in September, 1902, 375
Minnesota, September, 1902, 247
wins, 121
Minnesota State Veterinary Medical Association, 259
Missouri Veterinary Medical Association, the, 463, 590
Modern medicine, 577, 586
Modification in neurectomy, operative, 109
Molasses as a food for horses, 750
Monatsch. für Prakt. Thierk., 238, 297
Morphine, strychnine poisoning treated successfully with atropine and, 113
Multiple cerebral hemorrhages in the horse, 719
Mumps in the dog, 165
Munich. Mediz. Wochenschrift, 35
Municipal meat-inspection, 322
legislation, 6
Milk-inspection, some thoughts on, 337
Muscular origin, glycosuria of, 503

NATIONAL Livestock Association notes, 60
Zoological Park, report on the death of a hyena at the, 424
Native South Carolina cattle, Texas fever in, 81
Necrology, 46, 122, 177, 248, 314, 376, 444, 510, 587, 724, 773
Neglecting veterinary medicine, the penalty of, 508
Neiman, F. J., 329
Nelson, S. B., D.V.M., 45
Neoplasia exhibiting progressive malignancy, a condition of, 291
Nesom, G. E., D.V.M., 81
Neurectomy and flexor tenotomy for knuckling, 109
consolidated fracture of the second phalanx following, 764
in spavin, peroneal and tibial, 297
and flexor tenotomy for knuckling, median, 109
operative modification in, 109
Newborn, raw milk in marasmus and intestinal catarrh of the, 32
New Jersey, annual meeting of the Veterinary Medical Association of, 125
Board of Agriculture, 117
of Veterinary Medical Examiners, 399, 724
report of the semi-annual meeting of the Veterinary Medical Association of, 517
publications, 54, 130, 244, 588
New York American Veterinary College, commencement exercises, 530
County Veterinary Medical Association, 50
inspector's view, a greater, 378
Medical Journal, 509
Pasteur Institute, 172
State Veterinary Medical Society, 655
Niagara and Orleans County Veterinary Medical Society, 264, 529
Nighbert, E. M., D.V.S., 571
Nigra, poisoning by feeding on sinapis, 242
Nocard to Prof. Koch, reply of Prof. E., 1
Norton, J. C., V.S., 613, 648
Notes, 60, 131, 399, 464, 532, 604, 671, 727, 779
Nunn, Joshua A., 697
Nutrition of man, the improved respiration calorimeter for studying the, 186

OATS in the horse's ration, barley as a substitute for, 298
Observations on the treatment of scratches, mange, etc., 584
Obstetrical cases, 372
Obstetrics, 416
Œdema, 34
of the tongue, acute, 172
with carbolic acid, treatment of malignant, 301
Œsophagus, diagnosis of foreign bodies in the, 371
Œsterr. Monatsch. f. Thierheilk., 239, 297, 319
Official copy, reprint, 307
report of the proceedings of the fourteenth annual meeting of the Iowa State Veterinary Medical Association, 251
Ohio's splendid tribute to our late editor, Dr. Rush Shippen Huidekoper, 247
tribute to the memory of the late Dr. A. W. Clement, 247
Omentum, dermoid cyst of the great, 32
Ontario Veterinary Association, the, 58
College, 54, 726
commencement exercises, 380
Open joint, 208
Operative modification in neurectomy, 109
variation in arytenectomy, 297
Opotherapy with the juice of the sympathetic system, 113
Orchitis in a bull, chronic atrophic, 203
Orthodox method of treating parturient paresis and its etiology, a deviation from the, 405
Osteitis of founder, 110

Ottawa, American Veterinary Medical Association meeting, 726

Paisley, C. A., V S., 441
Palmer, H. F., D.V.S., 410
Parasites in the blood of epileptic persons, 423
Parturition cases, 211
Parasitic theories of cancer, 505
Parturient fever, simple treatment for, 774
  paresis and its etiology, a deviation from the orthodox method of treating, 405
  treatment of, 767
Passaic County Veterinary Medical Association, 521, 654, 728, 781
Pasteurized milk, calves and, 51
Pathogenesis of roaring, investigation of the, 469
Pathology, an atlas of clinical medicine, surgery, and, book review, 54
  of carcinoma, the, 580
  practical hints in, 228
Pathological study of ringbone and spavin, anatomo-, 133
Pearson, Leonard, B.S., V.M.D., 671
Peculiarity, an interesting, 377
Penalty of neglecting veterinary medicine, the, 508
Penis, amputation of a bull's, 302
Pennsylvania's contribution to the study of immunity to tuberculosis, 722
  State Board of Veterinary Medical Examiners, 192, 311, 651, 724
  Veterinary Medical Association, 47, 385, 463, 648, 664, 780
    committees, 1902–03, 458
    delegates, 1902–03, 459
Peroneal and tibial neurectomy in spavin, 297
Peroxide, clysters of hydrogen, 294
Personals, 62, 132, 197, 266, 332, 401, 466, 534, 602, 672, 732, 786
Petty, J. R., D.V.S., necrology, 724
Pfender, Charles A., 733
Phalanx, fracture of the glenoid fibrocartilage of the second, 296
Philippine Veterinary Medical Association, 197
Phillips, Walter S., V.S., 360
Phlegmasia dolens, or what? 419
Physicking in milk-fever cases, 399
Phytolacca poison in cattle, 439
Pig, alimentary intoxication of the, 577
  transmission of tuberculosis from man to the, 578
Piroplasma, canina, 33
Pistor, A. J., D.V.S., 706
Plan for controlling tuberculosis of cattle conservatively, a proposal for a new, 16
  for the disposal of tuberculous animals, 43
Pleura, abscess of the, 110
Pnuemonia, cold in etiogenesis of, 234
Pneumonic emphysema, the pathogenesis of quinine, 562
Points of a draught-horse, 31
Poisoning by feeding on sinapis nigra, 242
  treated successfully with atropine and morphine, strychnine, 113
Poison in sorghum, laboratory notes on, 704
Poll from foreign body, fistula of the, 719
Polyhydramnios, or dropsy of the amnion, 40
Pope, George W., D.V.S., secretary, 129, 521;
Potts, Miss Lulu M., marriage, 50
Practical hints in pathology, 228
President's address, 224, 407, 537
  Illinois State Veterinary Medical Association, 21
Presse Medicale, 295
Price, R., 261
Problems of milk fever, the unsolved, 230
Professional etiquette, 694
Progress in veterinary medicine, surgery, and its relation to public health, 104
Proper field of the American Veterinary Medical Association, what is the, 120
Properties and composition of the milk of the ass, 506
Prophett, Wm. H., D.V.S., necrology, 123
Proposition, Columella's, 175
Protargol in veterinary surgery, 269, 361
Publications, new, 130, 588
Puerperal mania in bovines, 571
Purpura hemorrhagica, 441
Pyæmia in the foal, 237
Pyramidon, 164
Pulmonary nodes in the lungs simulating glanders in a horse affected with summer wounds, 294

Quarter of a century's usefulness, 245
Quaker quality, 464

Rabies, 170, 214, 706
Rambaud, Geo. G., M.D., 172
Ranck, E. M., V.M.D., 57, 130, 343, 395, 463
Rayner, Moncure Robinson—frontispiece, 405
Rayner, Thos. B.—frontispiece, 405.
Rat, inoculation of cancer from man to the white, 504
Ravenel, M. P., M.D., 1, 65, 109, 138
Raw milk in marasmus and intestinal catarrh of the newborn, 32
Rayen, W. C., D.V.S., 625
Reagan, W. J., 729
Recueil de Medecine Veterinaire, 109, 164, 239, 295, 297, 319, 369, 371, 428, 504, 582
Reed, J. Hugo, V.S., 115
Reed, Jas. M., V. S., 416
Register, veterinary, 199, 267, 334, 404
Relative susceptibility to follicular mange, 365
Remedy for circumscribed follicular mange, 366
Reply of Prof. E. Nocard to Prof. Koch, 1
Reports of cases:
  Abnormal fœtus, an, 240
  Actinomycosis in a dog, 241
  Acute œdema of the tongue, 172
  Amnion, polyhydramnios, or dropsy of the, 40
  Amputation of bull's penis, 302
  Antitoxin in distemper, 116
  Calculi urethral, 115
  Cattle, an enzootic attack of chorea among, 37
  Chorea among cattle, an enzootic attack of, 37
  Creolin (Pearson) in arthritic synovitis, 116
  Distemper, antitoxin in, 116
  Dog, actinomycosis in a, 241
    a case of hæmatic filariasis in a, 507
  Dropsy of the amnion, polyhydramnios, or, 40
  Enzootic attack of chorea among cattle, 37
  Fœtus, an abnormal, 240
  Feeding on sinapis nigra, poisoning by, 242
  Impaction of the fourth fold of the large colon in a horse, 436
  Iodine treatment, tendinous synovial dilatations, 373
  Obstetrical cases, 272
  Observations on treatment of scratches, mange, etc., 584
  Œdema of the tongue, acute, 172
  Phytolacca poison in cattle, 439
  Poisoning by feeding on sinapis nigra, 242
  Polyhydramnios, or dropsy of the amnion, 40
  Purpura hemorrhagica, 441
  Rabies, 170
  Report of three interesting cases, a, 433

Reports of cases :
Rupture of the flexor tendons as a complication of azoturia, 303
Sinapis nigra, poisoning by feeding on, 242
Synovitis, creolin (Pearson) in arthritic, 116
Tetanus, 299
Treatment of malignant œdema with carbolic acid ; remarks upon "leeches" or bursattee, 301
Traumatic tetanus, 117
515
Urethral calculi, 115
Report of Committee on Animal Husbandry,
on Intelligence and Education, 447
on Legislation, 457
on Sanitary Science and Police, 513
Reports of committees, Pennsylvania State Veterinary Medical Association, 386
Report of three interesting cases, 433
of semi-annual meeting of the Veterinary Medical Assocatiion of New Jersey, 517
on the death of a hyena at the National Zoological Park, 424
Repp, John J., V.M.D., Sec., 58, 250, 299, 332, 436, 546, 593, 779
Resection of the trachea, suturing and, 33
Resident State secretaries, American Veterinary Medical Association, 1902–1903, 731
Resolutions, American Veterinary Medical Association, 599
Illinois Veterinary Medical and Surgical Association, 528
Iowa State Veterinary Medical Association, 321
Maryland State Veterinary Medical Association, 47
Minnesota State Veterinary Medical Association, 260
Pennsylvania State Veterinary Medical Association, 393
Respiration calorimeter for studying the nutrition of man, the improved, 186
Response by President Law, 658
Results of strict sanitary regulations in Arizona, 613
Retained afterbirth in cows, 620
Revue pharmac, 429
veterinaire, 32, 34, 111, 718, 764
Rinderpest, etiology of, 364
Ringbone and spavin, anatomo-pathological study of, 133
Rhoads, W. L., D.V.S., 512
Roaring, cricotomy for, 239
investigation of the pathogenesis of, 469
Roberts, Geo. H., Sec., 259
Roub, J. F., V.S., 242
Ruminants, gastro-intestinal strongylosis in, 504
Rules and regulations of the Board of Health of the city of Nashville, municipal meat-inspection legislation, with special reference to the law and the, 6
Runge, Werner, 126, 129
Rupture of the flexor tendons as a complication of azoturia, 303
Rutherford, Dr. J. G., 730

Saddlery and harness, injuries caused by ill-fitting, 697
Salicylas bismuth, 583
Sallade, Jas. W., V.S., 457
Salmon, D. E., Chief of Bureau of Animal Industry, 775, 778
Sanitary boards, interstate association of, 648
control of our periodic epizootics of anthrax, the value of co-operation in the, 484
work, 775
regulations in Arizona, results of, 613
Science and Police, report of the Committee on, 513
Sanitation, report of Committee on, 321
Saunders, Robert Jackson, V.S., 122
Scheile, Chas. V. S.—necrology, 376
Schuylkill Valley Veterinary Medical Association, 459, 785
Scratches, observations on the treatment of, 584
Scott, Geo. A., 329
Sec. de Biologie, 367
Secretaryships, 176
Selections, 51, 186, 577
Semaine Médicale, 766
Serum in anasarca, antistreptococcus, 238
of the horse in laparotomies, blood, 505
Seventeenth annual report of the Bureau of Animal Industry, new publication, 244
Sheep, vomeerziekte, or vomit-sickness of, 578
Shipley, L. U., D.V.S., 210
Shipley, T. A., 326
Shirari, Masanori, 53
Shoe boil, the etiology of, 688
Shorthorn breeders toward the tuberculin test, the attitude of the, 118
Shoulder lameness, treatment of "cold," 165
Sickness, horse, 356
Simpson, H. C., D.V.S., 203
Sinapis nigra, poisoning by feeding on, 242
Situs transversus in a bull, 288
Smith, D. King, M.D., 228
Smith, Ramsey W., B.S.C., M.B., C.M., 356
Smith, V. P., V.S., 40
Snakes, snake venoms, and antivenenes, 343
Society des Sc. Vet. de Lyon, 643
Society notes, 248, 785
Society Proceedings:
Allegheny County Veterinary Medical Association, 263, 782
Alumni Society of the Veterinary Department of the University of Pennsylvania 460
American Veterinary Medical Association, 130, 592, 652
Connecticut Veterinary Medical Association, 194, 729
Genesee Valley Medical Society, 193, 529
Illinois State Veterinary Medical Association, 320, 783
Veterinary and Surgical Association, the, 192, 527, 785
Indiana State Veterinary Association, 258
Interstate Association of Sanitary Boards, 648
Iowa State Veterinary Medical Association, 57, 251, 321
Keystone Veterinary Medical Association, 55, 129, 263
Massachusetts Veterinary Association, 398
Minnesota State Veterinary Medical Association, 259
Missouri Veterinary Medical Association, the, 463, 590
New Jersey Board of Veterinary Medical Examiners, 399
New York State Veterinary Medical Society, 655
Niagara and Orleans County Veterinary Medical Society, 264, 529
Ontario Veterinary Association, 58
Passaic County Veterinary Medical Association, 521, 664, 728, 781
Pennsylvania State Board of Veterinary Medical Examiners, 192, 651
Veterinary Medical Association, 385, 447, 458, 664, 780
Philippine Veterinary Medical Association, 197
Schuylkill Valley Veterinary Medical Association, 459, 785
Veterinary Association of Manitoba, 195
Medical Association of New Jersey, 125, 517, 728, 785
Wisconsin Society of Veterinary Graduates, 264, 663

Some thoughts on a city's milk supply, 352
on municipal milk-inspection, 337
diseases incidental to pregnancy and parturition in the cow, 630
experiments upon the immunization of cattle against tuberculosis, 673
Sorby, Harold, 184
Sorghum, laboratory notes on poison in, 704
South Africa, a trip to, 221
South American trypanosomatic disease of domestic animals, Voge's description of Mal de Caderas, a, 565
South Carolina cattle, Texas fever in native, 81
Spavin, anatomo-pathological study of ringbone and, 133
peroneal and tibial neurectomy in, 297
Spinal cord, experiments upon the excitability of the, 429
Stanton, D. C., V.S.—necrology, 376
State Board of Veterinary Examiners, 376
control of contagious diseases, 324
greetings, 647
Statter, G. P., V.S., M.D.V., 207
Stenosis of the duct in the teat of the cow, on, 574
Stevenson, H. A., M.D., 116
Stewart, H. L., M.D.C., 283
Stiles, Ch. Wardell, Ph.D., 565, 733
Stimson, J. Hayden, V.S., 46
Stocking, W. E., Secretary, 194, 529
Strabismus, editorial, 42
Strained tendons, insufflation of atmospheric air for, 296
Strongylosis in ruminants, gastro-intestinal, 504
Strychnine poisoning treated successfully with atropine and morphine, 113
Sudden deaths of gastric origin, 367
Summer wounds, pulmonary nodes in the lung simulating glanders in a horse affected with, 294
Surgery, protargol in veterinary, 361
Susceptibility to follicular mange, relative, 365
Sutton, D.S., V.S.—necrology, 376
Suturing and resection of the trachea, 33
Swain, S. H., V.S., 501
Swain, W. A., V.S., 193, 529
Sweetapple, C. H., V.S., 59, 184
Sympathetic system, opotherapy with the juice of the, 113
Syndactylism of the median digits in artiodactyla, 428
Synovial cyst of the knee in the horse, 111
dilatations, iodine treatment, tendinous, 373
Synovitis, creolin (Pearson) in arthritic, 116

TÆNIGEN, 601
Talbot, H. E., M.D.C., 287
Tapeworm, toxin of the, 644
Teat of the cow, on stenosis of the duct in the, 574
Telmosse, A. I., V.S., 178
Tendinous synovial dilatations, iodine treatment, 373
Tendons as a complication of azoturia, rupture of the flexor, 303
Tennessee, Texas fever and its relation to the livestock interests of, 625
Tetanus, 299
Texas fever and its relation to the livestock interests of Tennessee, 625
in native South Carolina cattle, 81
Theories of cancer, parasitic, 505
Therapeutic review, 34
use of lecithin, 236
Thirty-ninth annual meeting of the American Veterinary Medical Association, 592
Thomassen, M. H. J. P., 469
Thomsen, J., V.S., 208
Thoughts on municipal milk-inspection, some, 337
Thymol to expel whipworms from dogs, the failure of, 733
Tibial neurectomy in spavin, peroneal and, 297
Tick disease in the dog, 33
Timberman, J. H., V.S., 46
Tongue, acute œdema of the, 172
Torrance, F., D.V.S., 45, 197, 246
Torrance, W. J., 247
Tower, E. E., D.V.S., 250
Toxin of the tapeworm, 644
Trachea, suturing and resection of the, 33
Transmissibility of human tuberculosis to cattle, 367
Transmission of warts from the heifer to man, 33
Traumatic tetanus, 117
Treatment, antirabic, 239
of "cold" shoulder lameness, 165
of foot-and-mouth disease, 297
of hæmoglobinuria (azoturia), 111
of lameness, iodide of potash in the, 718
of malignant œdema with carbolic acid, 301
of parturient paresis, 767
of various diseases in dogs, clinical notes on the, 429
Treating parturient paresis and its etiology, a deviation from the orthodox method of, 405
Trials of the veterinary examining board, 287
Trichloride of iodine in distemper, 319
Trichorrhexis, 35
Trip to South Africa, a, 221
Trouble on the farm, 26
Trypanosoma in cattle, a new, 423
Tuberculin, 410
test, the attitude of the Dominion shorthorn breeders toward the, 118
of the farmer toward the, 90
Tubercle bacilli of bovine and human origin, comparative experiments on the pathogenic action for animals, especially those of the bovine species, of the, 167
bacillus from human and bovine sources, the comparative virulence of the, 65, 138
Tuberculosis from man to the pig, transmission of, 578
in cattle, the X-ray as an aid in the diagnosis of, 97
influence of alcohol upon the evolution of, 582
in the horse, 32
of cattle, Behring's recent announcement in regard to, 36
conservatively, a proposal of a new plan for controlling, 16
of the humerus, 32
Pennsylvania's contribution to the study of immunity to, 722
some experiments upon the immunization of cattle against, 671
to cattle, transmissibility of human, 367
to human beings, the final overthrow of Koch's theory respecting the non-communicability of bovine, 586
to man by accidental inoculation and experimental reinoculation of a calf, transmission of, 765
Tuberculous animals, a plan for the disposal of, 43
Tumor-like lesion in the lung of a horse caused by a blastomyces (Torula), 740
Tumors in the horse, frequency of, 111
Typhoid fever in a horse, 210

ULCERATIVE ano-vulvitis, external, 285
Ulcers, treatment of corneal, 718
United States Department of Agriculture, division of publications, January bulletin, 186

University of Pennsylvania, alumni society of the veterinary department of the, 460
commencement exercises of the veterinary department of the, 445
Unsolved problems of milk fever, the, 230
Urethral calculi, 115, 205
Urine, clitoridectomy for incontinence of, 164
Uses of asses' milk for infants, 35

VACCINATION against canine distemper, 506
Vaginal gestation, 238
Value of co-operation in the sanitary control of our periodic epizootics of anthrax, the, 484
of farm animals, 190
Van Es, L., M.D., V.S., 469
Various diseases in dogs, clinical notes on the treatment of, 429
Vein in the horse, jabot of the jugular, 35
Veterinarian, the, 578
Veterinary Association of Manitoba, 195
department of the University of Pennsylvania, alumni society of the, 460
commencement exercises of the, 445
dentistry, 757
inspection at the Boston horseshow, 189
journal, 581, 583, 641
medical association of New Jersey, 125, 517, 728, 785
medicine in its relation to public health, progress in, 104
the new era of, 605
the penalty of neglecting, 508
register, 199, 267, 334, 404
profession, anent "The Breeders' Gazette's" attitude toward the, 768
surgeon Desmond, 445
surgery, protargol in, 361
Virulence of the tubercle bacillus from human and bovine sources, the comparative, 65, 138
Voge's description of mal de caderas, a South American trypanosomatic disease of domestic animals, 565
Vomeerziekte, or vomit-sickness of sheep, 578

WALMER, Morris H., 178
Walrod, Geo. M., V.S., 302
War office, a joke in the, 52
Warts from heifer to man, transmission of, 33
Water drunk during digestion, action of, 370
Wattles, J. H., D.V.S., necrology, 773
Wattles, J. H., Jr., 773
Waugh, Jas. A., V.S., secretary, 263, 783
Weber, S. E., V.S., 45
Weight of the kidneys of the horse, the, 274
Welcome by Hon. Mr. Young, address of, 655
Welch, W. H., secretary, 321
Western veterinary college, 315
Where in 1902?, 42
Whipworms from dogs, the failure of thymol to expel, 733
White, G. R., D.V.S., 6, 439
Wien. klin. Wochens., 429
Williams, W. L., V.S., 620
Winchester, J. F., D.V.S., 537
Wisconsin Society of Veterinary Graduates, 264, 663
Wishes for the chief of the bureau of animal industry, 647
Wochenschrift für Thierheilkunde und Viehzucht, 432
Wyman, Wm. E. A., M.D., V.S., 28, 45, 269, 361, 405

X-RAY as an aid in the diagnosis of tuberculosis in cattle, the, 97

YOUNG, address of welcome by Hon. Mr., 655

ZEITSCHRIFT für Fleisch und Milch Hygiene, 169, 578

